# Molecular Mechanisms of Tumor Progression and New Therapeutic Strategies for Urological Cancers, 2nd Edition

**DOI:** 10.3390/ijms26136496

**Published:** 2025-07-05

**Authors:** Vicenç Ruiz de Porras

**Affiliations:** 1 GRET and Toxicology Unit, Department of Pharmacology, Toxicology and Therapeutic Chemistry, Faculty of Pharmacy and Food Sciences, University of Barcelona, 08028 Barcelona, Spain; vruizdeporras@ub.edu; Tel.: +34-606587010; 2 Badalona Applied Research Group in Oncology (B·ARGO), Catalan Institute of Oncology, Camí de les Escoles, s/n, 08916 Badalona, Spain; 3 CARE Program, Germans Trias i Pujol Research Institute (IGTP), Camí de les Escoles, s/n, 08916 Badalona, Spain

Urological cancers, including malignancies of the bladder, kidney, prostate, upper urinary tract, and penis, are among the most common cancers globally [1] and present significant heterogeneity in terms of clinical behavior, molecular biology, and therapeutic responsiveness [2]. Despite considerable advancements in local and systemic treatments, including immunotherapy and targeted therapies, the prognosis for many patients with advanced disease remains poor. Understanding the underlying molecular mechanisms of tumor progression and treatment resistance is essential to improve existing strategies and identify novel therapeutic targets.

In this second edition of the Special Issue “Molecular Mechanisms of Tumor Progression and New Therapeutic Strategies for Urological Cancers,” we feature seven contributions that shed light on critical aspects of tumor biology, biomarker discovery, and therapeutic innovation across various urological malignancies. These studies provide compelling evidence of the evolving landscape in precision oncology and liquid biopsy applications.

Non-invasive biomarkers based on liquid biopsy, capable of dynamically reflecting tumor behavior, are critical for real-time monitoring and therapeutic decision making [3]. In this context, two studies in the current Special Issue focus on circulating tumor-derived components as emerging tools in the management of urological tumors.

Carrasco et al. [4] explored a tumor-agnostic circulating tumor DNA (ctDNA) approach for monitoring patients with muscle-invasive bladder cancer (MIBC) after radical cystectomy (RC). By targeting common mutations (TERT and ATM) using droplet digital PCR, they demonstrated that ctDNA positivity was significantly associated with tumor progression at multiple timepoints. Importantly, ctDNA dynamics provided prognostic information superior to conventional imaging, aligning with prior reports that underscore the utility of ctDNA for early relapse detection [5,6] as well as for predicting immunotherapy efficacy in the adjuvant setting of MIBC [7]. This reinforces ctDNA as a minimally invasive and cost-effective strategy that can overcome the shortcomings of tissue-based biomarkers—including limited tissue availability, reliance on archival samples, and tumor heterogeneity—and is, as a result, increasingly used to guide treatment decisions [8].

In addition, Figols and colleagues [9] reviewed the role of tumor-educated platelets (TEPs) as a liquid biopsy source across urological tumors. TEPs, altered by interactions with tumors and their microenvironment, carry transcriptomic and proteomic signatures reflective of tumor burden and biology [10,11]. Their analysis highlighted TEPs’ diagnostic potential, especially in early cancer detection and non-invasive therapy monitoring, and support recent findings in other solid tumors, including non-small cell lung, glioblastoma, and pancreatic cancer [10,12,13].

On the other hand, treatment resistance remains a major challenge, particularly in bladder cancer, where cisplatin-based chemotherapy is still a cornerstone despite limited response rates [14]. Therefore, a better understanding of the mechanisms underlying therapy resistance is essential to developing novel treatment strategies and predicting drug response, ultimately advancing progress toward precision medicine. In this context, two articles in this Special Issue contribute to unraveling the molecular resistance mechanisms.

Elahi Najafi et al. [15] identified GABBR2 as a novel downstream effector of androgen receptor (AR) signaling, conferring cisplatin resistance in bladder cancer cells. The authors demonstrated that AR activation upregulates GABBR2, while its inhibition sensitizes cells to cisplatin. These findings suggest that targeting the AR-GABBR2 axis could reverse resistance, especially in AR-positive tumors. This is consistent with previous evidence that androgen signaling modulates bladder tumorigenesis, tumor progression, and therapy response, suggesting a potential role for androgen deprivation therapies in patients with bladder cancer [16,17].

In a related investigation, Vukovic et al. [18] explored the prognostic relevance of the IL-1β/IL-1RA axis in invasive bladder cancer. Their immunohistochemical analysis revealed that IL-1β expression correlated with aggressive pathological features but paradoxically conferred favorable survival. Moreover, IL-1β levels were positively associated with AKT and Ki-67 expression, implicating a role in autophagy and proliferation. These results reflect the complex dual role of inflammation in cancer, where pro-inflammatory cytokines may drive both tumor progression and anti-tumor immune responses [19].

In clear-cell renal cell carcinoma (ccRCC), extracellular matrix remodeling enzymes contribute to angiogenesis, invasiveness, and metastasis [20]. Młynarczyk et al. [21] compared MMP-14 and MMP-15 expression and activity in tumor and normal kidney tissues. They found that MMP-14 was significantly more active and expressed, particularly in higher-grade tumors, supporting its role in angiogenesis and invasiveness. These results reinforce earlier reports that link MMP-14 to disease progression and poor short-term prognosis in RCC and bladder cancer [22,23].

Although rare, penile squamous cell carcinoma (SCC) presents therapeutic challenges in advanced stages. Fahey et al. [24] described a case of metastatic penile SCC with a durable response to the antibody–drug conjugate, enfortumab vedotin, a human anti-nectin-4 antibody linked to the cytotoxic microtubule-disrupting agent monomethyl auristatin E [25]. Based on the results of the EV-301 phase III trial [26], enfortumab vedotin was approved by the FDA in 2019 for the treatment of patients with locally advanced or metastatic urothelial carcinoma who were previously treated with platinum-based chemotherapy and checkpoint inhibitor therapy. Interestingly, this report highlights the potential application of novel targeted agents beyond their initial indications and supports further investigation into Nectin-4 as a therapeutic target in penile cancer, consistent with expression patterns reported in urothelial and squamous histologies [25,27].

Prostate cancer (PCa), especially in its metastatic castration-resistant form (mCRPC), remains a challenging clinical entity that, despite significant advances in the treatment in the last decade, still carries a poor prognosis, with a median overall survival of approximately three years [28]. In this context, Porcel-Pastrana and colleagues [19] explored a combinatorial approach using hydroxytyrosol (HT), a phenolic compound from olive oil, and metformin, a widely used antidiabetic agent. Their in vitro data showed a synergistic inhibition of cell proliferation, migration, and tumorsphere formation across multiple PCa cell lines. Mechanistically, the combination suppressed key oncogenic pathways including MAPK, AKT, and TGF-β. These findings build upon prior preclinical evidence supporting the repurposing of metabolic regulators—primarily drugs used to treat diabetes, such as metformin, or to lower cholesterol, such as simvastatin—for cancer treatment and prevention [29,30,31].

Collectively, the contributions in this Special Issue reflect the diversity and depth of current research in urological oncology. From liquid biopsy technologies to resistance mechanisms and novel therapeutics, these studies point toward a future where disease management is increasingly personalized, biology-driven, and minimally invasive. We hope this compilation stimulates further research and translational efforts, ultimately improving outcomes for patients affected by these complex malignancies.
